# Beyond section Q: prioritizing nursing home residents for transition to the community

**DOI:** 10.1186/1472-6963-12-186

**Published:** 2012-07-03

**Authors:** Brant E Fries, Mary L James

**Affiliations:** 1 Institute of Gerontology, University of Michigan, 300 North Ingalls, Ann Arbor, MI, USA; 2 School of Public Health, University of Michigan, Ann Arbor, MI, USA; 3 Geriatric Research, Education, and Clinical Center, Ann Arbor VA Healthcare Center, Ann Arbor, MI, USA

## Abstract

**Background:**

Nursing Facility Transition (NFT) programs often rely on self-reported preference for discharge to the community, as indicated in the Minimum Data Set (MDS) Section Q, to identify program participants. We examined other characteristics of long-stay residents discharged from nursing facilities by NFT programs, to “flag” similar individuals for outreach in the Money Follows the Person (MFP) initiative.

**Methods:**

Three states identified persons who transitioned between 2001 and 2009 with the assistance of a NFT or MFP program. These were used to locate each participant’s MDS 2.0 assessment just prior to discharge and to create a control sample of non-transitioned residents. Logistic regression and Automatic Interactions Detection were used to compare the two groups.

**Results:**

Although there was considerable variation across states in transitionees’ characteristics, a derived “Q + Index” was highly effective in identifying persons similar to those that states had previously transitioned. The Index displays high sensitivity (86.5%) and specificity (78.7%) and identifies 28.3% of all long-stayers for follow-up. The Index can be cross-walked to MDS 3.0 items.

**Conclusions:**

The Q + Index, applied to MDS 3.0 assessments, can identify a population closely resembling persons who have transitioned in the past. Given the US Government’s mandate that states consider all transition requests and the limited staffing available at local contact agencies to address such referrals, this algorithm can also be used to prioritize among persons seeking assistance from local contact agencies and MFP providers.

## Background

A key feature of states’ long term care “re-balancing” efforts has been the establishment and expansion of programs to assist nursing facility (NF) residents to return to less expensive and more integrated community settings. Collectively known as nursing facility transition (NFT) programs, 44 states are now engaged in the Centers for Medicare and Medicaid Services’ (CMS) $1.75B Money Follows the Person (MFP) initiative, targeted at long-stay residents, and a number of states have dedicated increasingly scarce general funds to similar NFT efforts [1] for both the short-and long-stay nursing facility population.

While program goals vary across states and funding sources, it is widely recognized that to be considered a success, NFT programs must identify residents unable to transition in the absence of assistance, rather than assisting those who would have otherwise returned to the community without outside help [1]. Section Q of the National Resident Assessment Instrument/Minimum Data Set (MDS) 2.0 contains two items that record a resident’s interest in returning to the community (Q1a) and whether family members were supportive of this preference (Q1b). Despite efforts by CMS to make this information more readily accessible, early NFT programs often struggled to identify appropriate transition candidates [2,3]. State goals generally remain modest: among the first 31 MFP programs funded by the federal government, less than 1% of the total institutional population was targeted for transition, and several states have reduced their initial goals as they encountered a variety of program implementation roadblocks [4]. These problems have not diminished after the October 2010 debut of the new MDS 3.0.

In light of these early NFT implementation experiences, the current project addressed two hypotheses. First, we expected that each state would have unique program goals and thus would likely target different transitionee populations. By comparing transitionee characteristics across several states, all states could become aware of different possible target populations that could enlarge and broaden their NFT initiatives. Second, we hypothesized that specific characteristics of NFT program participants would distinguish them from individuals who remain in NFs. We sought an algorithm to “flag” NF residents who would be contacted to discuss potential community transition. Properly designed, this algorithm would target a relatively small percentage of all residents yet would successfully identify a very large percent of those who were actually transitioned in the past.

This project was unique in its multi-state comparison of NFT participant characteristics. Our intent was to enable states to improve NFT targeting strategies and thereby improve the use of scarce fiscal resources earmarked for transition activities.

## Methods

### Data

The study used data from three states with NFT programs – Michigan, Arkansas, and Illinois – and required two primary components. First, data were needed to identify the NF residents the three states actually transitioned to the community, including name, social security number, birth date, and the date of transition. Second, full data were needed that described both the transitionees and each state’s NF population on a common measurement metric. These were provided by the MDS Version 2.0 that was nationally mandated to be completed on every NF resident, regardless of payment source, at standardized intervals. The scientific basis of the MDS is well established [5]. In each state, we attempted to find assessments in the MDS archives for each NFT transitionee. The three state programs were:

Arkansas: The Arkansas sample was comprised of persons enrolling in the Division of Aging and Adult Services, Arkansas Department of Health and Human Services PASSAGES program from CY2001 to 2004 [6]. The program was carried out at the local level by four Area Agencies on Aging and three Centers for Independent Living. A total of 118 Medicaid eligible transitionees were enrolled. Among these, we matched 112 individuals (94.9%) with Arkansas MDS data.

Illinois: The Illinois sample came from two different transition programs, one housed in the Illinois Department on Aging and the other in the Department of Rehabilitation Services (DORS) [7]. Data were provided by the Illinois Department of Healthcare & Family Services for 359 transitionees from CY2002 to 2008. Of these, we matched 326 individuals (90.8%) with MDS records.

Michigan: The Michigan Department of Community Health provided a list of 313 Medicaid-eligible individuals who transitioned as part of the state’s 2008–2009 MFP initiative [8] and enrolled in the MI Choice waiver. We matched 304 individuals (97%) with MDS data.

For each state, we first identified all MDS assessments for each NFT resident. Although an assessment was unlikely performed just at the time of transition, we approximated the characteristics of a resident at this time by using the most recent assessment prior to the transition date. If this assessment was not a full assessment (i.e., it was a truncated quarterly assessment), we completed the missing variables using information from a prior full assessment, following the procedure used by CMS to generate Resident Profile Table (RPT) records (see [9] for example). As items not on the quarterly assessment are known to vary less frequently over time, and a substantial change in resident status is supposed to trigger a “Significant Change” assessment, the RPT represents a good approximation of the resident’s characteristics at the time of the last assessment before transition.

We also developed a control sample of residents not transitioned. The sample was selected at random from a population of all 148,877 assessments available; if a quarterly assessment was selected, it was completed using the RPT method. For every NFT person, regardless of how the stay was funded, we randomly chose 100 non-NFT resident assessments in the same state and calendar year, being sure that no resident was selected in more than one year.

The NFT and controls were merged into a single analytic data set and all personal identifiers were removed (e.g., date of birth was transformed into years of age). Day of stay was calculated at the time of the assessment, as we wished to have a compatible measure for both NFT and non-NFT residents.

Combining the three states’ data, the ”full sample” database represented 742 transitionees, with a control sample of 74,200. However, here we report analyses on a subset of individuals, specifically persons with days of NF stay (at the time of assessment) of 90 days or more. This decision was made so that our targeting algorithm would identify the priority population identified in the MFP enabling legislation. Congress encouraged states to focus on the “long-stay” population by creating an attractive financial incentive: states participating in MFP can earn additional federal matching funds for a period of one year for all Medicaid home and community-based services provided to this target group.

The final analytic database represented 327 long-stay individuals (1.9% of the long-stay population across the three states) who transitioned, and a control sample of 17,476 residents with stays of at least 90 days. The control sample included both Medicaid eligible and privately funded individuals, as our previous work has demonstrated that the two long-stay sub-groups are very similar in their clinical characteristics. Also included in the control sample were 2,602 individuals (3.5% of those in the initial database) for whom information on admission date was missing; we included these persons as our experience has demonstrated that it is longer staying residents for whom admission dates are not recorded. This analytic database, as a whole or divided into subsamples for the three individual states, was used for all model building and testing. The full sample of 148,877 individuals was used in the final steps to estimate prevalence, sensitivity, and specificity.

### Measures

The MDS is a broad instrument; each full assessment includes more than 400 items in eighteen diverse domains. With such a large number of MDS items and the relatively small number of NFT residents, it was necessary to choose a more limited set of variables to describe transitioning and non-transitioning residents. We based our choices on our work with states over the past decade to refine profiles of their nursing facility populations for use in policy decisions; we also relied on our previous research and clinical insight. Thus, we used the following in our analyses:

*Scales:* A number of scales have been designed to summarize domains of the MDS, i.e., algorithms that compound multiple MDS items into a more reliable and valid single measure. We employed the following:

○: *Cognitive Performance Scale:* a unified seven-category rating of cognitive function based on memory impairment, level of consciousness, and executive function [10]. The CPS has been shown to be highly correlated with the Mini Mental State Examination. To further reduce the number of variables (and statistical degrees of freedom), the CPS was trifurcated into three ranges: 0–1 (intact), 2–4 (impaired), 5–6 (severely impaired).

○: *Activities of Daily Living Hierarchy:* a rating, ranging from 0 (independent) to 6 (total dependence), of ADL functional impairment [11]. The scale is calculated according to the sequence of ADL loss with early loss ADLs (such as dressing) receiving a lower score compared to late loss ADLs (such as eating and bed mobility).

○: *Depression Rating Scale:* a screen for clinical depression based on seven MDS items detailing mood problems [12]. The DRS has been validated using the Hamilton Depression Rating Scale and the Cornell Scale for Depression. It has a range of 0–14, with higher scores indicating higher levels of depression.

○: *Communication Scale:* a rating of communication ability combining ability to understand and to be understood by others. This scale has a range of 0–6, with increasing values indicating poorer communication ability.

○: *Psychosocial Well-being Scale:* a unified system of assessing resident happiness, sense of control, social involvement, and satisfaction [5].

○: *Behavior and Severe Behavior Scales*: a composite of behavior problems exhibited during the past seven days, including wandering, verbally abusive behavior, physically abusive behavior, socially inappropriate behavior, and resisting care. The Behavior Scale counts the number of these (0–5) occurring at least once in the period; the Severe Behavior Scale counts the number (0–5) of these behaviors that occurred daily.

○: *Pain Scale:* examines the frequency and intensity of pain shown by an individual. It has been validated against the Visual Analogue Scale [13].

○: *Resource Utilization Groups, Version 3 (RUG-III):* a case-mix system that places NF residents into groups based on intensity of care needs [14]. Associated with each of the 44 groups is a Case Mix Index (CMI) representing the relative nursing and therapy costs of residents in that group. For the purposes here, the CMIs were split into six bands, with break-points determined by inspecting the values and the distribution of residents across the 44 groups.

Other resident characteristics, including:

○: Age (in years, at the time of assessment)

○: Clinical characteristics: includes diagnoses (e.g., Parkinson’s disease, bipolar disorder), disabilities (e.g., hemiplegia, paraplegia, or quadriplegia), 90-day improvement/decline in cognition, sensory problems (vision, hearing), terminal illness, pressure ulcers, etc.

○: Discharge potential (Section Q). Two yes/no items on the MDS measure important aspects of the person’s interest in and ability to be discharged. One describes whether the “resident expresses/indicates preference to return to the community” (item Q1a), while the second records if “resident has a support person who is positive towards discharge” (Q1b). In both cases, missing responses were coded to “no.”

Service variables, including:

○: Day of NF stay (at the time of the last assessment before the person’s transition date, as described earlier)

○: Use of physical restraints. Although clinical “service” variables are avoided in many applications to prevent purposeful under- or over-reporting, we included this variable to address the possibility that it could “stand in” for others that described the person’s condition.

○: Whether the person was admitted to the NF from his or her home, rather than a hospital, other NF, etc.

Finally, we had two preliminary measures to identify NFT residents that came from our prior study of the Arkansas PASSAGES project [15]. There we used Automatic Interactions Detection (AID) [16] to identify two groups of NF residents like those who had transitioned to the community. The first (“Arkansas Narrow”), identifies persons who “look like” PASSAGES participants - only 1.5% of persons in Arkansas NFs. Over the course of a year, this approach would identify for consideration approximately 250 of the more than 16,700 Medicaid eligible individuals who utilize Arkansas NFs annually. The sensitivity of this approach was 62%; in other words, this strategy would correctly identify the individuals resembling PASSAGES participants almost two thirds of the time. The specificity of this approach was 98.5%; such a strategy would incorrectly identify individuals as resembling non-PASSAGES individuals only 1.5% of the time. The second, broader measure (“Arkansas Broad”) identifies all NF residents *except* those meeting the criteria for the group containing the majority of non-PASSAGES participants. This approach had a sensitivity of 92% and a specificity of 83%. Over the course of a year it would identify for evaluation approximately 2,800 Medicaid-eligible residents, or 16.8% of all Medicaid eligible residents in Arkansas.

The full list of variables used in the analysis is listed in Table 1.

**Table 1:** Prevalence of selected resident characteristics for samples used in analysis: short- vs. long-stay residents in three states combined; controls vs. nursing facility transfer (NFT) for long-stay residents in three states combined; and long-stay NFT residents in three individual states

**Resident Characteristics**	**Three states, combined**					**Individual states LOS**^**1**^ **> =90,NFT**			
	**Full sample**^**2**^		**LOS > =90**						
	**LOS < 90 days**	**LOS > =90 day**	**Controls**	**NFT**	**Significance**^**3**^	**Arkansas**	**Illinois**	**Michigan**	**Significance**^**3**^
Number of observations	57,464	17,476	17,149	327		56	91	180	
Age category					**				*
Under 55 years	4.9%	6.6%	6.2%	29.1%		50.9%	33.0%	20.6%	
55 to 64 years	6.4%	7.0%	6.6%	23.0%		20.0%	26.4%	22.2%	
65 to 74 years	17.4%	13.4%	13.3%	21.5%		12.7%	20.9%	24.4%	
75 to 84 years	36.9%	31.2%	31.5%	18.4%		9.1%	11.0%	25.0%	
85 or more years	34.5%	41.8%	42.5%	8.0%		7.3%	8.8%	7.8%	
ADL Hierarchy category					**				**
Independent (0, 1)	9.4%	15.0%	14.7%	34.3%		42.9%	56.0%	20.6%	
Somewhat dependent (2, 3, 4)	66.1%	50.9%	50.9%	55.1%		50.0%	39.6%	64.4%	
Dependent	24.5%	34.0%	34.5%	10.7%		7.1%	4.4%	15.0%	
Task segmentation	58.3%	57.7%	58.2%	34.6%	**	26.8%	48.4%	30.0%	**
Quadriplegia/hemiplegia/paraplegia	6.2%	9.0%	8.8%	21.1%	**	28.6%	8.8%	25.0%	*
CPS category					**				**
Intact (0, 1)	52.3%	28.6%	27.8%	66.7%		83.9%	74.7%	57.2%	
Impaired (2, 3, 4)	42.5%	57.2%	57.6%	31.8%		16.1%	25.3%	40.0%	
Severely impaired (5, 6)	5.2%	14.3%	14.5%	1.5%		0.0%	0.0%	2.8%	
Cognitive improvement	0.7%	2.0%	1.9%	3.7%	*	7.1%	2.2%	3.3%	
Cognitive deterioration	4.3%	7.3%	7.3%	2.8%	*	0.0%	7.7%	1.1%	*
Communication problem	32.4%	55.8%	56.5%	19.9%	**	14.3%	11.0%	26.1%	*
Involved in activities >1/3 of time	90.5%	84.7%	84.5%	96.0%	**	92.9%	94.5%	97.8%	
Behavior problem (any)	14.5%	34.4%	34.6%	25.1%	*	32.1%	33.0%	18.9%	*
Severe behavior problem	2.4%	7.8%	7.3%	2.5%	*	1.8%	5.5%	1.1%	
Depression (DRS > =2)	19.8%	31.0%	30.9%	36.1%	*	26.8%	45.1%	34.4%	
Bipolar disease	1.9%	3.6%	3.5%	6.1%	*	7.1%	7.7%	5.0%	
Schizophrenia	2.0%	8.4%	8.5%	6.7%		3.6%	15.4%	3.3%	*
Hearing problem	9.5%	18.9%	19.0%	10.1%	*	10.7%	6.6%	11.7%	
Vision problem	21.5%	34.6%	34.9%	19.3%	**	8.9%	19.8%	2.2%	
Bowel incontinence	22.6%	39.4%	39.9%	16.5%	*	17.9%	7.7%	20.6%	*
Bladder incontinence	24.1%	43.7%	44.1%	22.9%	**	16.1%	11.0%	31.1%	*
Dehydrated	0.6%	0.5%	0.5%	0.0%		0.0%	0.0%	0.0%	
Pain (severe)	4.0%	2.0%	2.0%	3.7%	*	5.4%	5.5%	2.2%	
Pressure ulcer stage > =2	19.0%	16.2%	16.3%	10.1%	*	10.7%	9.9%	10.0%	
Terminal illness	1.3%	2.0%	2.0%	1.5%		0.0%	0.0%	2.8%	
Diabetes	32.3%	30.4%	30.2%	38.5%	*	16.1%	31.0009%	48.9%	**
Cancer diagnosis	4.5%	4.3%	4.3%	2.5%	*	1.8%	5.5%	1.1%	
Parkinson disease	1.9%	4.4%	4.4%	3.4%		1.8%	1.1%	5.0%	
Any cardiac diagnosis	57.9%	64.8%	64.7%	69.2%		54.7%	62.4%	76.7%	*
Physically restrained	2.4%	8.6%	8.8%	1.5%	**	3.6%	2.2%	0.6%	
RUG-III CMI category					**				**
.46 to .57	2.7%	15.4%	15.1%	32.4%		53.6%	47.3%	18.3%	
.58 to .72	2.4%	16.3%	16.5%	8.0%		3.6%	6.6%	10.0%	
.73 to .91	16.5%	20.4%	20.3%	29.1%		16.1%	23.1%	36.1%	
.92 to 1.09	50.4%	23.6%	23.7%	19.0%		19.6%	16.5%	20.0%	
1.10 to 1.25	5.7%	5.0%	5.0%	3.4%		1.8%	2.2%	4.4%	
1.26 to 1.70	22.4%	19.2%	19.4%	8.3%		5.4%	4.4%	11.1%	
LOS category					**				
Under 60 days	96.4%	NA	NA	NA		NA	NA	NA	
60 to 89 days	3.7%	NA	NA	NA		NA	NA	NA	
90 to 179 days	NA	13.2%	13.1%	19.7%		20.0%	25.3%	17.2%	
180 to 364 days	NA	19.1%	18.8%	32.4%		24.0%	32.9%	34.4%	
365 to 729 days	NA	24.7%	24.6%	29.8%		30.0%	30.4%	29.4%	
730 days or more	NA	43.0%	43.6%	18.1%		26.0%	11.4%	18.9%	
Admitted from home	4.0%	7.3%	7.3%	5.8%		8.9%	7.7%	3.9%	
Residents prefers return to community (Q1a)	78.2%	19.8%	18.1%	63.9%	**	55.4%	55.0%	71.1%	*
Support person positive about discharge (Q1b)	29.5%	3.6%	3.1%	30.3%	**	25.0%	16.5%	38.9%	*
Arkansas-narrow criteria^4^	0.3%	6.1%	5.3%	43.4%	**	67.9%	42.9%	36.1%	**
Arkansas-broad criteria^4^	12.9%	23.8%	23.0%	64.2%	**	91.1%	70.3%	52.8%	**

Notes:

1) LOS = length of stay until assessment.

2) Full sample includes 100 controls for every NFT observation. All differences in the full 3-state sample between LOS < 90 day and LOS > =90 were significant at p < .05, except for Task Segmentation and Cancer.

3) Significance: * = p < .05; ** = p < .0001.

4) Arkansas-narrow and Arkansas-broad criteria not used in logistic regressions.

## Methods

Our analysis was conducted in several steps.

We first compared profiles of each state’s NFT and non-NFT population, using the measures described above. Differences across the different states on specific characteristics were tested using comparative statistics of means (z-statistics) and distributions (chi-squared statistics).

Second, we considered bivariate statistics to identify which of the many measures would be most associated with persons who were transitioned. This could potentially allow us to reduce the list of measures modeled.

Third, we used stepwise logistic regression models to identify resident characteristics (including the two service measures listed earlier) associated with the dependent variable of interest – a dichotomous variable representing those individuals transitioned as compared to the control sample of non-transitioning NF residents. In this step, we excluded the two multivariate composite “Arkansas” measures – we compare these later. We developed models both for the three-state analytic sample and for each individual state, hypothesizing that each state program would target, at least in part, different types of residents.

Using the results of the final logistic regression for the three-state sample, we developed a “Q + Index” so named to reflect the addition of variables beyond those found in section Q of the MDS. The Q + Index summarizes the multiple predictors in a simple and practical way. It was calculated by:

reversing all items with odds ratios under 1.00 (for example, a characteristic with an odds ratio of 0.581 was changed to the “absence of the characteristic” which had an odds ratio of 1/0.581, or 1.721);

weighting each of the items with the (adjusted) odds ratios in excess of 5.0 as a “3” and all of those with odds ratios between 2 and 5 as a “2”, compared to all of the other items weighted as “1”;

summing the scores.

While this approach cannot be expected to produce the optimal Index, it represented a balance between the statistical evidence and simplicity. We then checked the created Index, again using logistic regression, to see how well it predicted NFT compared to the full logistic model.

We also tried AID as an alternate approach to developing a predictor algorithm. AID provides groups of observations that, taken together, identify the subpopulation of interest.

Finally, we evaluated the fit of various models by contrasting their sensitivity and specificity. The tradeoffs between the two were examined visually through a Receiver Operating Characteristic (ROC) curve, which plots sensitivity on the vertical axis against the false positive rate (i.e., 1 minus the specificity). Good alternative models would be closer to the upper left corner of the graph. We also compared models using the c-statistic, which represents the area under the ROC curve; values in excess of 0.7 are considered indicators of good fit.

Analyses were performed using SAS Version 9.1.3. AID is available as part of the SAS Enterprise Miner package, Version 5.3 [17].

This study and its protocols were approved by the Institutional Review Board of the University of Michigan as secondary data analysis.

## Results

Individuals who had days of stay of less than 90 days were significantly and substantially different on virtually every measure considered when contrasted with individuals evaluated in the rest of the analyses, i.e., those with days of stay of 90 days or more (see Table 1). Shorter-stayers were more cognitively intact (53% had intact cognition, compared to 29% for longer-stayers), and had fewer medical problems such as hearing loss (10% vs. 19%), vision loss (22% vs. 35%), bladder incontinence (24% vs. 44%), depression (20% vs. 31%), etc. This confirmed our decision to focus our analyses on the longer-stayers, viz. the mandated target of MFP programs.

The average age of the residents with days of stay over 90 days – our analytic sample – was 78.8 years, with 42% over the age of 85 years, and 65.6% female (not shown). Within this sample, the NFT sub-sample was, as expected, significantly skewed to the younger and less disabled compared to all other longer-staying residents: only 8% of NFTs were over the age of 85 years (vs. 43% of other longer-staying residents), 11% (vs. 35%) were dependent in ADL, and 2% (vs. 15%) were cognitively severely impaired (see Table 1). In fact, on almost all of the measures chosen for this study, the NFT population was less disabled. The exceptions included only quadriplegia/hemiplegia/paraplegia, depression, bipolar disease, severe pain, and diabetes. For schizophrenia, dehydration, terminal illness, Parkinson’s disease, cardiac conditions, and whether admitted from home, the differences in prevalence were not statistically significant. However, we decided to retain all the variables, including those without significant relationship to NFT status, as there were only a few measures that could be eliminated and most had potentially substantial clinical reason to be considered.

We also saw substantial differences in the characteristics of transitionees in each of the three states (see Table 1). For example, Michigan’s NFT population had significantly higher proportions of individuals with communication problems, cognitive impairment (moderate or more severe impairment, i.e., CPS of 2 or more), bladder incontinence, diabetes, a cardiac diagnosis, and the most dependency in ADLs, while Illinois had the highest proportion of individuals needing task segmentation and having schizophrenia, and the lowest proportion of those who had quadriplegia, hemiplegia, or paraplegia. These inter-state differences were most often not mirrored in the control sample of the three states (i.e., in non-transitionees with days of stay of 90 days or more – results not shown).

The primary focus of the research was to determine which characteristics described the residents who were able to transition. Altogether, 16 characteristics were independently predictive of NFT in a logistic regression, even after controlling for others (Table 2). The characteristics with the highest odds ratios for transition (i.e., over 2 or under 0.5) were age (specifically under age 84, and especially under age 75); quadriplegia/hemiplegia/paraplegia, involvement in activities at least 1/3 of the time; in the least resource-intense groups under the RUG-III system; and an expressed interest in returning to the community, along with the absence of three additional characteristics: schizophrenia, the need for task segmentation, severely impaired cognition (CPS of 5–6), and a stay of over 2 years. The model had a fairly robust fit, with a c-statistic of 0.908.

**Table 2:** Variables statistically significant in explaining NFT status in logistic regressions, for residents with length of stay 90 days or more, in three individual states and combined

**Variable**	**Three states (N = 17,476)**		**Arkansas (N = 4,693)**		**Illinois (N = 11,204)**		**Michigan (N = 1,578)**	
	**OR**	**Signif**	**OR**	**Signif**	**OR**	**Signif**	**OR**	**Signif**
**c-statistic**	**0.908**		**0.949**		**0.903**		**0.875**	
Age category		<.0001		<.0001		<.0001		<.0001
Under 55 years	12.606	<.0001	71.088	<.0001	3.449	0.2970	13.630	0.0003
55 to 64 years	12.137	<.0001	20.551	0.0035	7.336	<.0001	12.880	<.0001
65 to 74 years	7.197	0.0028	7.154	0.9699	4.289	0.0379	9.414	0.0088
75 to 84 years	2.708	<.0001	1.665	0.0014	1.220	0.0060	3.873	0.0188
85 or more years (reference)								
ADL Hierarchy category		0.0013				0.0018		
Independent (0, 1)	1.333	0.7779			6.721	0.0004		
Somewhat dependent (2, 3, 4)	1.991	0.0003			3.746	0.2740		
Dependent (reference)								
Task segmentation	0.363	<.0001			0.543	0.0236	0.619	0.0204
Quadriplegia/hemiplegia/paraplegia	2.832	<.0001	2.940	0.0199			3.048	<.0001
CPS category		0.0480		0.0006				
Intact (0, 1) (reference)								
Impaired (2, 3, 4)	0.707	0.7483	See note					
Severely impaired (5, 6)	0.423	0.1569	See note					
Cognitive deterioration					3.024	0.0111		
Communication problem	0.581	0.0019			0.219	0.0001	0.470	0.0010
Involved in activities >1/3 of time	2.691	0.0050						
Behavior problem (any)	0.687	0.0206						
Depression (DRS > =2)	1.481	0.0065			1.693	0.0336	1.720	0.0114
Schizophrenia	0.320	<.0001						
Hearing problem	1.537	0.0467					2.137	0.0189
Diabetes			0.267	0.0048				
Any cardiac diagnosis	1.541	0.0033						
RUG-III CMI category		0.0005		0.0095				<.0001
.46 to .57 (reference)								
.58 to .72	0.762	0.2785	0.256	0.7959			0.561	0.0530
.73 to .91	0.775	0.0753	0.392	0.5629			0.427	0.1530
.92 to 1.09	0.433	0.0188	0.195	0.3389			0.178	0.0021
1.10 to 1.25	0.597	0.9415	0.423	0.7158			0.209	0.2315
1.26 to 1.70	0.338	0.0032	0.097	0.1193			0.143	0.0004
LOS category		<.0001				<.0001		<.0001
Under 60 days		NA						
60 to 89 days		NA						
90 to 179 days	2.189	0.4501			8.980	0.0007	0.724	0.0006
180 to 364 days	2.931	0.0002			7.229	0.0058	1.801	0.0738
365 to 729 days	2.427	0.0634			4.727	0.5503	2.572	0.0002
730 days or more (reference)								
Residents prefers return to community (Q1a)	3.732	<.0001	14.946	<.0001	2.222	0.0028	2.237	0.0004
Support person positive about discharge (Q1b)	2.938	<.0001			2.074	0.0329	3.217	<.0001

Note: For the Arkansas sample, there were no NFT observations within the CPS Severely Impaired category. Thus, the CPS coefficients cannot be estimated. OR = Odds Ratio; Signf = Significance.

These logistic results were not, however, mirrored in the logistic regressions run on each state’s data individually, reflecting their different NFT targeting practices. While all three models had good statistical fit, only age and preference to return to the community were consistently seen as predictors across all three states’ logistic regressions, although each model picked up a selection of the other variables significant in the combined-state model.

The logistic regression results for the full three-state sample were used as described earlier to build a “Q + Index” that could identify the relative likelihood that a person was like individuals who actually were transitioned to the community.

Of the 16 statistically significant variables, three were eliminated on clinical grounds, as it was unreasonable to associate them with increased likelihood of transition: depression, hearing impairment, and cardiac conditions. Further analysis, not displayed here, showed that the inclusion of these three variables provides only minimally superior performance, not sufficiently large to rule out their statistical significance as more than a spurious result.

**Figure 1:**
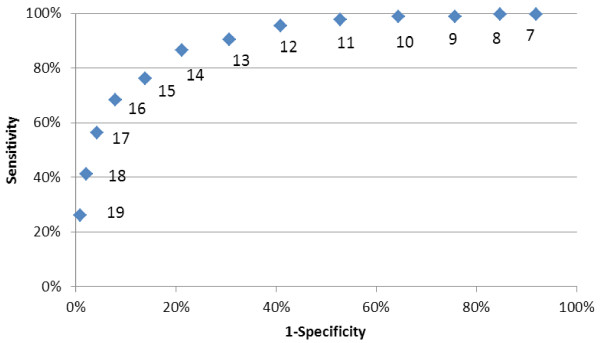
Worksheet for Computation of the Q+ Index (MDS 2.0 variables and values).

The calculation of the Index is displayed in Figure 1. By construction, it can take on values from 0 to 24. For transitionees, the mean Index score was 16.6, compared to 10.9 for those not transitioned.

**Figure 2:**
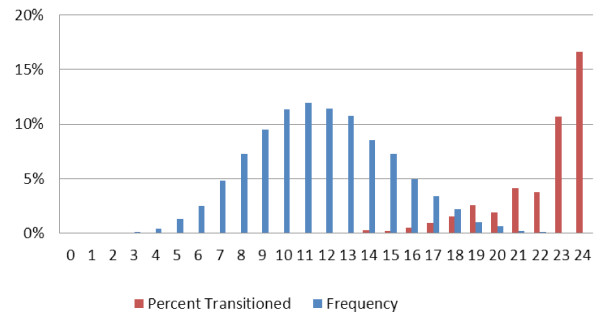
ROC Curve for Q+ Index Thresholds (Three State Data, N= 17,476, including 327 NFT).

A simple use of the Q + Index is with a single “threshold” value: those who exceed this value are more carefully considered for potential transition. By inspection, thresholds of 14 or 15 are the best tradeoffs between sensitivity and specificity, i.e., those closest to the upper left corner of the ROC curve in Figure 2. Of these two very comparable options, we tentatively opted for the less restrictive criterion (threshold 14 or more) to increase the likelihood of identifying successful NFT candidates.

**Figure 3:**
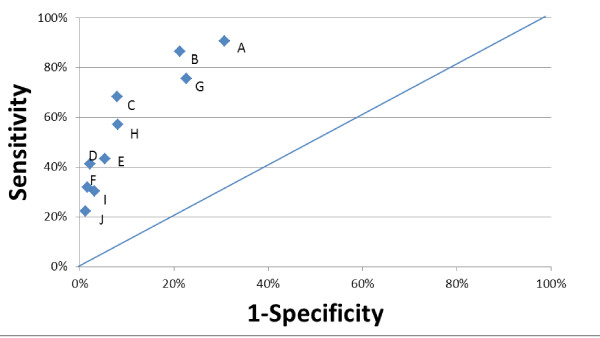
Percentage NFT Transitionees and Frequency, by Q+ Index Value (Full Three State Data, n = 148,866, including 327 NFT).

An alternate approach is to use the Index as a numeric prioritization, where persons with higher scores represent those most likely to be similar to previously transitionees. Returning to the original full database of all 148,877 assessments, almost no transitionees are found when the Q + Index takes on values less than 10, but this increases to 10.7% and 16.7% of all long-stay residents with Index values of 23 and 24 (Figure 3).

The AID analysis found that the two summary measures – the Q + Index described above and the Arkansas “Narrow” criterion from our pilot study-- dominated all other predictive variables. In fact, over all the statistical models run both for the individual three states and the three states combined, AID identified only five variables useful outside the Index itself: resident expresses/indicates preference to return to the community (Q1a), resident has a support person who is positive toward discharge (Q1b), the cognitive performance scale (CPS), age, and RUG-III CMI.

Ten models, combining these variables in various configurations, were run on the three-state long-stay database. The four best involved only the Q +  Index and are displayed in Table 3, which also provides the specificity and sensitivity of these dichotomous (yes/no) models. For example, Model A triggered those residents who had a Q + Index score of 13 or greater, for 31.8% (5565/17476) of the sample; all others were considered not triggered. This criterion had a sensitivity of 90.5% and specificity of 69.3%.

**Table 3:** **Comparing alternate dichotomous indicators of NFT (Three-state sample with LOS > =90 days**^**1**^**)**

**Models**	**Target group**				**Sensitivity**	**Specificity**	**c**	**Label in Figure**4
	**Total triggered**	**% All observations**	**Non-NFT**	**NFT**				
Q + Index > =13	5565	31.8%	5269	296	90.5%	69.3%	0.799	A
Q + Index > =14	3928	22.5%	3645	283	86.5%	78.7%	0.826	B
Q + Index > =16	1579	9.0%	1356	223	68.2%	92.1%	0.801	C
Q + Index > =18	503	2.9%	368	135	41.3%	97.9%	0.696	D

^1^ n = 17,476, including 327 NFT.

**Figure 4:**
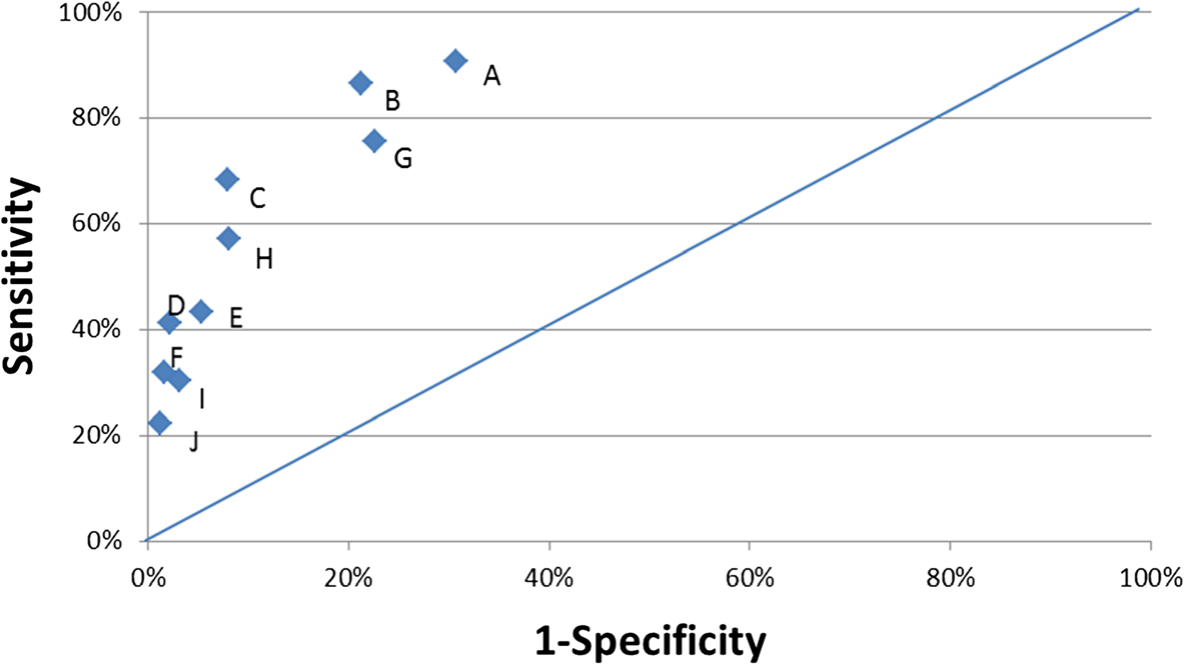
Sensitivity and Specificity for Alternate Dichotomous Indicators of NFT (3-State Data, n = 17,476, including 327 NFT).

The comparisons of sensitivity and specificity of the 10 models run on the 3-state combined data – Models A through J – are displayed in a ROC-like plot (Figure 4). From this, it can be seen that the two best models in terms of combined sensitivity and specificity use the Q + Index with a threshold of 14 (Model B) or 16 (Model C). Using the c-statistic of a logistic regression as the criterion, Model B (c = 0.826, vs. 0.801) was superior.

We also tested these same models in each individual state, and found that the Q + Index with a threshold of 14 uniformly performed the best or next-best (results not shown).

Finally, we applied this algorithm (with a threshold of 14) to all residents across the three states with at least 90 days of stay. A total of 28.3% of all assessments would be triggered, including 86.5% of the NFT assessments; overall the algorithm achieved a sensitivity of 86.5% and specificity of 78.7%.

The derivation work was performed using historical information from the MDS 2.0. As of October 2010, MDS 3.0 became the mandated NF assessment system. Of the 13 MDS 2.0 items and scales in the Q + Index, 10 are reasonably mapped to MDS 3.0 items, viz. all except Task Segmentation, Involvement in Activities, and that a support person is positive about discharge. When run on the MDS 2.0 omitting these three items, and with slightly adjusted weights, the “MDS 3.0 Q + Index” ranges from 0 to 18. With the threshold best set at 11, the algorithm triggers a slightly lower estimated percentage of all long-stay SNF residents: 25.5%. It correlates well with the original Q + Index (r = .925), has slightly higher sensitivity (88.1% vs. 86.5%), and slightly lower specificity (74.7% vs. 78.7%) and c-statistic (.814 vs. .826).

## Discussion

The analysis of three states’ NFT participants demonstrated that each state differed in the individuals identified for transition. This allowed us to identify several NFT targeting algorithms. The best, as measured by a combination of specificity, sensitivity, and logistic regression c-statistics, was a “Q + Index” based on 13 resident characteristics and scales used to target residents scoring 14 or more. This Index and threshold was the best both across the three states, and in two of the three individual states; for the three-state sample it demonstrated superior sensitivity (86.5%) and specificity (78.7%). The crosswalk to MDS 3.0 produces an Index with similar characteristics, but direct testing on future NFT participants using actual MDS 3.0 data remains to be accomplished.

The 3.0 Q + Index has immediate and practical utility. It can easily be run on a state’s MDS data to identify likely transition candidates, as it requires no additional data collection and could be automated with a computer algorithm. Clearly, the Index would add value to Section Q of the MDS; our analysis indicated that the Section Q information by itself was not particularly useful to identify the individuals who actually transitioned. One implementation possibility would combine Section Q with the Index. A state could use Section Q to identify individuals who wish to return home and use the Index to prioritize future transitionees, giving the highest priority to persons with the highest Index scores. Alternately, a state could use the Q + Index to identify persons who resemble previously transitioned individuals, and prioritize based on Section Q responses.

Other results also have take-home messages useful to policymakers and clinicians. Of compelling interest is that the characteristics of our final NFT sample, selected to mirror the long-stay target group for the federally funded MFP demonstration, were very different from the short stay NF population. Substantial differences were seen among short- and long-stayers in nearly all (30 of 32) the clinical characteristics we tested, a finding with implications for both the design and timing of NFT targeting efforts. Recent federal policy intent is to ask the MDS 3.0 Section Q at each assessment and to refer all individuals who want to go home to a “local contact agency” unless they expressly reject such a referral. Included in these referrals will be a large number of people with less than 90 days of NF stay; not only are such individuals numerous, but our data also show that they are very likely (78.2%) to indicate a preference to return to the community. Most of these people have previously been discharged without special efforts. However, under the new Section Q policies, local contact agencies must now sift through all of these referrals to identify the more rare long stay individuals requiring outside assistance to transition. Anecdotally, states report large back-logs of referrals at the local contact agencies, which are often minimally staffed. Our data suggest that a more efficient approach would delay referrals to local contact agencies until the first quarterly reassessment, when the majority of individuals who could return home without assistance (the short stayers) have already done so. Among long-stayers in our study that actually transitioned, 63.9% had indicated a preference to return home, as opposed to 18.1% of the overall long stay population. This delay in referrals would enable the local contact agencies to focus limited staffing resources on the target population identified in the MFP statute.

We also found that while the characteristics of the long stay populations across the three states were fairly similar, the persons transitioned were quite heterogeneous. This suggests that the target group for NFT may be more diverse than estimated in the emerging literature. For instance, Mor and colleagues proposed that the most likely group for community based care would be those with the least impairment [18]. While our study affirmed that lower-acuity individuals (as measured by RUG-III) were more prevalent among transitionees than among those who remained in the NF (32.4% vs. 15.1%), we also found that some relatively rare subsets of long stayers with severe ADL impairment – those with hemiplegia, paraplegia, and quadriplegia – were 2.8 times more likely to be transitioned than persons without these conditions. Similarly, while across the three states we found that persons with impaired cognition were less likely to transition than those who were cognitively intact, nearly 40% of Michigan’s NFT sample was cognitively impaired. This finding counters assertions that persons with cognitive difficulties are not an appropriate target group [19]. Finally, age played a major role in predicting transition; over half the transitionees were under age 65, although persons in this age group comprised only 12.8% of the longer-stay sample.

We recognize several important limitations to this research. First, our study had a small sample size; we would have preferred to include more NFT individuals and more states. In particular, a limited number of NFT individuals made it impossible to reserve a subsample for validation testing. Even across only three states, there were substantial differences in the characteristics of NFT participants; it remains a question how well our three-state results would generalize to the whole nation. Second, our study included only those who actually returned to the community. Enlarging the sample to include persons who tried but failed in their transition experience would enable better understanding of the relative weight of individual characteristics or extrinsic factors (e.g., type of transition assistance, service availability, housing needs) in achieving transition. Third, we lacked follow-up data on transitionee outcomes. Anecdotally, we know that some people who transition do return to institutional settings, either for a transient stay or on a permanent basis. Factoring in the follow-up status of individuals would potentially clarify the characteristics associated with long-term community placement, thus providing another avenue to hone the Q + Index and maximize the return on investment necessary to assist transitionees. This issue was beyond the scope of our current effort, but could help to establish evidence-based measures regarding which timeframes or outcomes constitute NFT “success.” Fourth, analysis is needed to assure that the results here, derived on MDS 2.0, also apply to MDS 3.0. Finally, we focused here on residents with days of stay of 90 days or more; additional analysis would be needed to refine the algorithm for a short-stay, non-MFP population.

## Conclusions

Overall, our findings demonstrate that the Q + Index is a useful tool to identify and prioritize long-stay residents “likely” for transition, even across states whose transitioned population appeared quite different at the outset. Further, our findings underscore the value of state engagement in collaborative analysis to improve program effectiveness and efficiency.

## Competing interests

The authors declare that they have no competing interests.

## Authors’ contributions

BEF, performed all the statistical analysis for the study, wrote the sections on Methods and Results, and participated in the final editing. ML. J., planned and coordinated the study, collaborated with the states involved, wrote the Introduction and the Discussion section, and participated in the final editing. All authors read and approved the final manuscript.

## Pre-publication history

The pre-publication history for this paper can be accessed here:

http://www.biomedcentral.com/1472-6963/12/186/prepub
